# Job Insecurity and Subsequent Actual Turnover: Rumination as a Valid Explanation?

**DOI:** 10.3389/fpsyg.2020.00712

**Published:** 2020-04-16

**Authors:** Anne Richter, Tinne Vander Elst, Hans De Witte

**Affiliations:** ^1^Department of Learning, Informatics, Management and Ethics, Karolinska Institutet, Stockholm, Sweden; ^2^Center for Epidemiology and Community Medicine, Stockholm County Council, Stockholm, Sweden; ^3^Faculty of Psychology and Educational Sciences, KU Leuven, Leuven, Belgium; ^4^IDEWE – External Service for Prevention and Protection at Work, Heverlee, Belgium; ^5^Optentia, North West University, Vanderbijlpark, South Africa

**Keywords:** job insecurity, rumination about job insecurity, turnover, mediation, longitudinal design

## Abstract

Job insecurity is a work stressor with many negative consequences for the individual as well as the organization. However, currently, little is known about why job insecurity is related to these outcomes. In the present study, actual turnover was investigated as a possible consequence of job insecurity. Additionally, rumination about a possible job loss (i.e., the act of intensified thinking about the future of the job) was investigated as an explanatory mechanism. Relationships were tested using longitudinal data from a sample of 699 Belgian employees. Results of structural equation modeling analyses show that job insecurity was related to turnover 1 year later. This relationship was mediated by rumination about job insecurity. Actual turnover was investigated over time as a potential consequence of job insecurity, compared to many studies that used turnover intention as a proxy to predict actual turnover. Moreover, a job insecurity-specific mechanism—namely, rumination about job insecurity—was studied, which increased our understanding of how job insecurity develops into its consequences.

## Introduction

Job insecurity, defined as the perceived threat of job loss and the worries related to that threat (De Witte, 2005), has been related to a variety of negative consequences with implications for both the individual employee and the organization. One of these organization-related consequences is actual turnover, that is, job change. Even though turnover has mostly been studied indirectly through turnover intentions (e.g., Berntson et al., 2010; Emberland and Rundmo, 2010; Staufenbiel and König, 2010), turnover intentions do not always result in actual turnover (Tett and Meyer, 1993) and might thus not be the best way to investigate job change. There is little evidence that employees experiencing job insecurity actually leave their job in the long run (e.g., Arnold and Feldman, 1982; Blau, 2007), and the existing evidence is rather inconsistent. In addition, we have comparably little understanding of the explanatory mechanisms underlying the job insecurity–turnover relationship; why do employees change jobs when they experience job insecurity?

In this study, we introduce rumination about job insecurity as a new potential mediating mechanism in the relationship between job insecurity and actual turnover. Rumination about job insecurity impairs the successful unwinding from work and hence drains individuals of energy. Based on the Conservation of Resources (COR; Hobfoll, 2001) theory, we predict that job insecurity depletes employees’ resources through repetitive thoughts and worries about the future of their job. In turn, this rumination about job insecurity may result in actual voluntary turnover as a way to withdraw from the stressful job situation. Rumination about job insecurity may also predict involuntary turnover: individuals who dwell on the future of their job and experience associated stress complaints, such as ill health, cannot perform in their work role as expected of them, which increases their chances of involuntarily losing their job.

This study contributes to the existing job insecurity literature in several ways. First, we study the relationship between job insecurity and actual turnover in the future, an objective outcome that has major consequences for organizations but has received rather limited attention in previous research. Previous studies investigating turnover intention as a proxy for actual turnover have taken on the assumption that individuals’ intentions are strongly related to subsequent behavior, which is not automatically the case (Cohen et al., 2015). Therefore, this study contributes by highlighting an overlooked outcome of job insecurity. Second, we introduce rumination about job insecurity as a new mechanism that could explain the relationship between job insecurity and turnover. Instead of focusing on general rumination, we focus on a job insecurity-specific mechanism, namely work-related rumination about job insecurity. This refers to the act of repetitively thinking about and dwelling on the insecure future of the job. By studying the relationship between job insecurity and rumination about job insecurity, we highlight the process of stress experiences (cf. loss spirals in COR; Hobfoll, 2001). Most theoretical explanations for the job insecurity–outcome relationship that have been investigated (e.g., perceived control; Vander Elst et al., 2014c) (e.g., the breach of the psychological contract; De Cuyper and De Witte, 2006) derive from cognitive frameworks (e.g., appraisal theory; Lazarus and Folkman, 1984) and social exchange theory (Cropanzano and Mitchell, 2005) but do not account for the emotional and behavioral intensification of the job insecurity experience. Third, we used a two-wave repeated measurement design in which we studied the *prospective* relationship between job insecurity and rumination about job insecurity on the one hand and actual turnover on the other hand. As such, it was possible to highlight effects of job insecurity and rumination about job insecurity over time. Fourth, by offering evidence of a new explanatory mechanism underlying the relationship between job insecurity and actual turnover, this study provides policy makers with some practical guidelines to prevent actual turnover in times of uncertainty. This is important, as scholars have found that the best workers are the ones who leave the organization first (Greenhalgh and Rosenblatt, 1984).

### Job Insecurity and Turnover

Building on COR (Hobfoll, 1989, 2001), we predict that job insecurity is related to actual turnover over time. According to this theory, individuals’ well-being depends on their resource pool. Resources are entities that have an instrumental value for individuals, such as objects (e.g., a house or car), conditions (e.g., being employed), personal resources (e.g., skills), and energies (e.g., mental and physical energy). The maintenance or increase of resources is associated with well-being. A threat of resource loss or an actual decline in resources, on the other hand, is related to negative consequences such as strain (Hobfoll, 1989, 2001). When employees experience job insecurity, one of their most essential resources, namely being employed, is under threat, which may deplete employees’ resources even further. Employees may try to protect their remaining resources by withdrawing from the stressful situation. One way of withdrawing from a job-insecure situation is changing jobs (Maertz and Campion, 2004; Blau, 2007; Filipkowski and Johnson, 2008). In addition, job insecurity may lead to turnover as a result of an involuntary job loss. According to COR, previous resource losses can make individuals more susceptible to future resource loss. Through the experience of job insecurity, employees’ performance at work might be impaired (Cheng and Chan, 2008), as job-insecure employees invest energy in dealing with the threat of potentially losing their job and experience stress complaints. Over time, job insecurity may therefore result in losing the job involuntarily as employees can no longer fulfill the organization’s expectations (Iverson and Pullman, 2000).

In most studies, the job insecurity-turnover relationship was examined indirectly, via the proxy of turnover intentions (e.g., Berntson et al., 2010; Emberland and Rundmo, 2010; Staufenbiel and König, 2010). In these studies, job insecurity was associated with higher levels of turnover intentions (Sverke et al., 2002; for meta-analytic results, see Cheng and Chan, 2008). The general assumption is that attitudes such as intention to leave the organization result in actual behaviors such as leaving the organization. Even though there is a consensus that there is a positive relationship between turnover intention and actual turnover, the strength of this relationship has been found to vary (Cohen et al., 2015), implying that high levels of turnover intention do not always result in actual turnover. Studies on the relationship between job insecurity and actual turnover are, however, scarce (Iverson and Pullman, 2000; Maertz and Campion, 2004; but see e.g., Blau, 2007; Filipkowski and Johnson, 2008). Using occupational turnover data, Blau (2007), for instance, found a significant positive relationship between job insecurity and voluntary organizational turnover. However, Iverson and Pullman (2000) did not establish a relationship of job insecurity with turnover, either voluntary or involuntary.

Based on our theoretical arguments and the indirect evidence regarding turnover intentions, we predict the following:

*Hypothesis 1*: Job insecurity at T1 is positively related to turnover at T2.

### Rumination About Job Insecurity as a Mediating Mechanism

Even though there is some evidence of the relationship between job insecurity and turnover, we are not aware of studies investigating the explanatory mechanisms underlying this relationship. We propose rumination about job insecurity as an explanatory factor underlying this relationship (cf. Niesen et al., 2014). Rumination about job insecurity represents a specific type of work-related rumination: Employees repetitively think about and dwell on the potential loss of their job. Rumination about job insecurity can clearly be distinguished from job insecurity (i.e., the *fear* of losing the current job), as it refers to the *act* of more severe and intensified thinking about the future of the job that has a recurring and repetitive nature. The act of ruminating about job insecurity implies that employees are more strongly anticipating job loss in the future and thus perceive an inevitable threat to the job (Lyubomirsky et al., 1999). Rumination about job insecurity can therefore be perceived as a more intensified and behavioral consequence of feelings of job insecurity. As such, it can be considered a stress reaction to the initial perceived stressor of job insecurity (cf. Sverke et al., 2002).

So far, rumination has dominantly been investigated in clinical and health psychology, and it is known to have an important role in the etiology of mental disorders, such as anxiety disorder or depression (Lyubomirsky et al., 1998; Mellings and Alden, 2000). As a consequence, rumination has mainly been studied in a rather general way, which leaves many questions open regarding what individuals ruminate about and how rumination applies to the work context. In this study, we investigated acts of rumination focusing on the possibility of losing the job in the future (“rumination about job insecurity”), which may be a highly relevant mechanism for understanding how job insecurity results in certain negative consequences.

Theoretically, the relationship between job insecurity and rumination about job insecurity can be understood building on COR (Hobfoll, 1989, 2001). This theory states that the threat of a resource loss evokes stress reactions and attempts to protect the valued resources. Job insecurity, indicative of the threat to the job, may result in stress reactions and attempts to find solutions in the eventual case of job loss, which may be reflected in rumination about a possible job loss in the future (Hartley et al., 1991; Richter et al., 2013). Rumination can thus be conceived as an indicator of the intensification of the job insecurity experience. For instance, rumination, which is evoked by a problematic situation, is perceived as a dynamic process that depends on the certainty of a situation and the quantity of information individuals have access to. The more indications individuals have that something negative is going to happen, the more they ruminate about it (Lyubomirsky et al., 1999). When job-insecure individuals experience an increase in the chances of a potential job loss, the initial feelings of job insecurity may develop into the more intensified cognitive activity of ruminating about job insecurity. This corresponds to the literature on rumination in general, in which rumination has been found to be triggered by problematic situations in which a discrepancy between the actual (e.g., perceived job insecurity) and the preferred situation (e.g., preferred job security) is perceived (Zeigarnik, 1983; Martin et al., 1993; Smith and Alloy, 2010). We may therefore predict that experiences of job insecurity are related to rumination about job insecurity.

The act of rumination may further deplete employees’ resources. After all, constantly and repetitively thinking about the possibility of job loss in the future and its implications is stressful and may evoke strain. Rumination has, for example, been related to delay in recovery, mental and physical symptoms (e.g., Hazlett and Haynes, 1992; Sarason et al., 1996), anxiety disorder and depression, and low feelings of control and negative self-evaluations (Lyubomirsky et al., 2003). According to COR (Hobfoll, 1998, 2001), employees experiencing strain might remove themselves or withdraw from the aversive situation as a way to reduce their exposure to the aversive environment and to protect their remaining resources (Taris et al., 2001). Accordingly, we predict that rumination about job insecurity may lead to voluntary turnover over time as a way for employees to protect themselves. Employees who ruminate and dwell on the possible loss of their job in the future are severely drained of energy, which might lead to a conscious decision to change jobs in order to feel better and stop depleting their resources. Similarly, previous studies have, for instance, found that job strain is a strong predictor of turnover in the long run and that individuals try to shift from their demanding job situation to a job with better conditions and less strain in order to preserve their resources (Drake and Tadama, 1996; Todd and Deecry-Schmitt, 1996; Wright and Cropanzano, 1998; de Croon et al., 2004).

Rumination may also lead to involuntary turnover. Employees who are drained of energy and cannot completely focus on their job will experience difficulties in meeting their employer’s expectations and may therefore be more vulnerable to be dismissed and forced to change jobs over time. Previous studies have demonstrated that performance is negatively affected by work stressors, such as job insecurity, and that employee performance is negatively related to involuntary turnover (Bycio et al., 1990). Similarly, employees who ruminate about job insecurity may no longer engage in their job to the extent that is required and might therefore be more vulnerable to be dismissed. Employees who ruminate about job insecurity might thus be forced to look for another job due to involuntary job loss and to change jobs to a higher degree.

**FIGURE 1:**
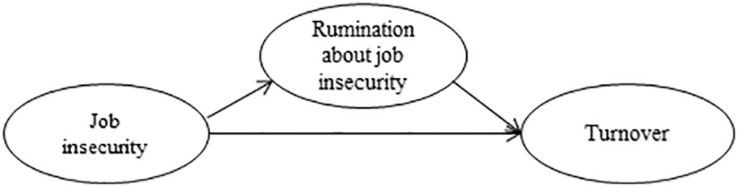
Theoretical model of job insecurity and turnover through rumination about job insecurity.

Job insecurity may thus result in rumination about job insecurity, which in turn may predict actual turnover over time (see Figure 1 for a representation of our theoretical model). We therefore hypothesize the following:

*Hypothesis 2*: Rumination about job insecurity at T1 mediates the relationship between job insecurity at T1 and turnover at T2.

## Materials and Methods

### Data Collection and Respondents

The sample consisted of readers of the Flemish human resources magazine Vacature^1^ who participated in a large online study on stress and rumination in the Belgian workforce. In December 2005 (T1), Vacature called for participation in an open survey via the newsletter and the website. Respondents were informed about the study aim, the voluntary nature of the survey, and the confidential treatment of the data. Therefore, entering the survey was considered agreement to the terms (“informed consent”). After a strict data cleaning procedure (e.g., based on e-mail address and the combination of background characteristics such as employment status to exclude unemployed persons and respondents who participated in the survey multiple times), complete data regarding the study variables were obtained from 9,518 employees. Respondents who expressed interest in the study results (*N* = 7,643) were invited to participate in a follow-up study 1 year later (December 2006; T2). In total, 869 individuals responded at T2 (longitudinal response of 11%). After omitting respondents with missing data, we arrived at a final sample of 699 employees with full information on the variables of interest at T1 and T2.

The final sample consisted of 403 male (58%) and 296 female employees (42%). The majority had a permanent contract (77%). Eleven percent of the employees were under 25 years old, 46% were between 25 and 34 years old, 24% between 35 and 44 years, and 15% between 45 and 54 years. Employees older than 55 years were in the minority (4%). Thirty-nine percent of the employees had a university degree, and 71% of the sample was Dutch (versus French) speaking.

A dropout analysis, using logistic regression, was conducted to investigate potential differences between respondents who participated at both time points and those who only participated at T1. We specifically investigated whether dropout at T2 was predicted by five background characteristics (i.e., age, gender, contract, language, and education), as well as job insecurity and rumination about job insecurity at T1. The results indicated that, overall, drop-out at T2 could be predicted based on the predictors in the model, χ^2^(7) = 29,18, *p* = 0.00. In particular, younger employees dropped out more at T2 compared to older employees, which was found to be the only difference between those who only participated at T1 and those who participated at both T1 and T2.

### Measurements

Both Dutch and French versions of the measurements were used in this study. The original Dutch measurements were translated into French by professional translators. Afterward, bilingual researchers in the domain of work and organizational psychology checked for the semantic, conceptual, and normative equivalence of the Dutch and French measurements, using the back-translation procedure.

*Job insecurity* was measured with one item (“I worry about the future of my job”), deriving from the Short Inventory on Stress and Well-Being (Vander Elst et al., 2011). This item presents the core of the affective job insecurity construct and closely resembles items from other validated and commonly used job insecurity scales (Hellgren et al., 1999; e.g., Vander Elst et al., 2014b). The respondents could indicate their answers on a scale from 1 (*strongly disagree*) to 7 (*strongly agree*). High values indicate higher concerns about potential job loss.

*Rumination about job insecurity* was measured with three items (“I ruminate about … that the future of my job is not ensured,” “… that I will be fired,” “… whether I will be able to keep my job”) from Hermans et al. (2006), which tapped into rumination about the possibility of losing the current job. The reliability was satisfactory for both time points with α_T__1_ = 0.87 and α_T__2_ = 0.85 (Nunnally and Bernstein, 1994). The respondents were asked to rate these items on a scale from 1 (*not at all*) to 5 (*very much*). Higher values on this scale indicate more rumination about issues concerning job insecurity.

*Actual turnover* was measured with one dichotomous item at T2, where individuals indicated whether they had changed to a job at another organization during the last year (1 = yes, 0 = no).

Five covariates were included in the analyses, which were found to influence job insecurity and its outcomes in previous research (De Witte et al., 2015) or reflected the sample characteristics: age (years), gender (1 = female, 0 = male), education (1 = university degree, 0 = no university degree), contract type (1 = permanent contract, 0 = temporary contract), and language (1 = French, 0 = Dutch) were taken into consideration.

### Statistical Analyses

Structural equation modeling was conducted by means of Mplus 8.4. For the preliminary analyses (i.e., confirmatory factor analysis), maximum-likelihood (ML) estimation could be applied because the analyzed data were measured on a five-point Likert scale. However, when testing the hypotheses, weighted least squares means and variance adjusted (*WLSMV*) estimation was applied to account for the dichotomous nature of our outcome variable. To ensure that the data were normally distributed, we screened the kurtosis and skewness values of the study variables. We did not find kurtosis values greater than 10 or skewness values greater than 3 (Weston and Gore, 2006; Kline, 2010). Additionally, no variables were highly correlated (*r* > 0.85; Weston and Gore, 2006), suggesting that multicollinearity was not likely.

Model fit was evaluated using the comparative fit index (CFI; Bentler, 1990), the standardized root mean-square residual (SRMR), and the root mean square error of approximation (RMSEA; Steiger, 1990). For the CFI value, the traditional cut-off criterion is > 0.90 (Marsh, 2007; Kline, 2010), whereas the more strict criterion is > 0.95 (Hu and Bentler, 1999). For RMSEA, values lower than 0.08 indicate acceptable fit (Marsh, 2007; Kline, 2010), whereas values under 0.06 point at good model fit (Hu and Bentler, 1999). Regarding SRMR, values smaller than 0.10 indicate acceptable fit. To compare nested models, the chi-square difference test was used (Weston and Gore, 2006). When the dichotomous outcome variable turnover was included in the model, the robust chi-square difference test was used instead (Satorra, 2000; Asparouhov and Muthén, 2006).

#### Preliminary Analyses

To evaluate the discriminant validity of job insecurity and rumination about job insecurity, confirmatory factor analyses (CFA) were conducted. Two models were compared. Model 1, a four-factor model, consisted of rumination about job insecurity at T1 and rumination about job insecurity at T2, measured by three original items each, and job insecurity at T1 and T2 was measured with one item each. In Model 2, a two-factor model, job insecurity and rumination about job insecurity were measured as one construct at T1 and T2; hence, four items at T1 and T2 measured a combined construct of job insecurity and rumination about job insecurity. In line with recommendations by Bollen (1989), the item-specific measurement errors were allowed to correlate over time to account for the systematic method variance associated with each indicator. These two models were compared to see whether job insecurity and rumination about job insecurity acted as two separate constructs. Factor loadings and the covariance between the factors were inspected to investigate whether job insecurity and rumination about job insecurity were different constructs.

In addition, we examined whether the measurement model of rumination about job insecurity-the only variable that was measured with multiple indicators-was the same over time, using longitudinal CFA (Brown, 2006). In this two-factor model, the auto-regressions between the constructs at T1 and T2 were included. The item-specific measurement errors were allowed to correlate over time to account for the systematic method variance associated with each indicator (Bollen, 1989). An unconstrained model was tested and compared with a model in which the factor loadings for the specific items of the rumination scale were constrained to be equal over time. Afterward, the intercepts were set to be invariant over time. Factorial invariance is a requirement for interpreting potential temporal changes as true changes and not as changes in the measurement model (Brown, 2006), allowing for more reliable conclusions regarding the relationships between the investigated variables. In this study, we gathered data among Dutch- and French-speaking employees, therefore using a Dutch and a French translation of the measurements. To ensure that we measured the same in both language groups, we investigated whether the measurement model of rumination about job insecurity-the only variable that was measured with multiple indicators-was the same across the Dutch- and French-speaking employees using multiple group CFAs. Increasingly constrained models (models with freely estimated parameters, invariant factor loadings, invariant indicator intercept) were tested and compared using the chi-square difference test.

#### Test of Hypotheses

To investigate Hypothesis 1, a model specifying the path from job insecurity at T1 to actual turnover at T2 was estimated. This model included an auto-regression path from job insecurity at T1 to job insecurity at T2, but not for turnover, which was only measured at T2.

To investigate Hypothesis 2, both a full mediation model and a partial indirect model were tested. The total indirect model included paths from job insecurity at T1 to rumination about job insecurity at T1 and from rumination about job insecurity at T1 to actual turnover at T2. The partial mediation model also included a direct path from job insecurity at T1 to turnover at T2. The auto-regression paths (i.e., from job insecurity at T1 to job insecurity at T2; from rumination about job insecurity at T1 to rumination about job insecurity at T2) were included in these models, with an exception for turnover, which was only measured at T2. The best model in terms of model fit and parsimony was selected, and the indirect effect was calculated. Bias-corrected bootstrapping with 1000 bootstrap samples was applied to calculate 95% confidence intervals for the indirect effect (Preacher and Hayes, 2008; Little, 2013). We controlled for the five background variables in all analyses.

## Results

### Descriptive Results

Table 1 shows the means, standard deviations, and inter-correlations for all variables, as well as the reliability of the rumination scale. In accordance with our expectations, job insecurity at T1 was positively related to rumination about job insecurity and actual turnover at T2, and rumination about job insecurity at T1 was positively related to turnover at T2. The only covariate that was related to either job insecurity, rumination about job insecurity, or turnover at T2 was contract: those having a permanent contract reported lower job insecurity and lower rumination about job insecurity and were less likely to change jobs at T2.

**TABLE 1:** Means, standard deviations, reliability (Cronbach’s alpha coefficient in parentheses), and inter-correlation (*N* = 699).

	*M* (*SD*)	1	2	3	4	5	6	7	8	9	10
(1) Gender T1	0.58 (0.49)	–									
(2) Age T1	2.56 (0.99)	−0.26*	–								
(3) University education T1	0.28 (0.48)	0.02	−0.10*	–							
(4) Permanent contract T1	0.77 (0.41)	−0.10*	0.18*	–0.06	–						
(5) Language T1	0.28 (0.45)	–0.03	−0.07*	0.03	−0.07*	–					
(6) Job insecurity T1^a^	3.92 (1.88)	0.02	0.01	–0.05	−0.15*	0.13*	–				
(7) Job insecurity T2^a^	3.78 (1.80)	0.05	0.05	–0.03	−0.08*	0.13*	0.40*	–			
(8) Rumination T1^b^	1.87 (1.08)	0.00	0.04	–0.04	−0.22*	0.08*	0.60*	0.34*	(0.87)		
(9) Rumination T2^b^	1.77 (1.00)	0.05	0.07	0.00	−0.12*	0.06	0.38*	0.61*	0.51*	(0.85)	
(10) Turnover T2	0.16 (0.37)	–0.01	–0.07	–0.06	−0.10*	–0.02	0.16*	–0.07	0.17*	–0.03	–

*p < 0.05, calculations conducted in SPSS. Gender: 1 = female, 0 = male; Age: 1 = younger than 25, 2 = 25–34, 3 = 35–44, 4 = 45–54, 5 = 55 and older; University education: 1 = yes, 0 = no, Permanent contract: 1 = yes, 0 = no; Language: 1 = French, 0 = Dutch; Turnover: 1 = yes, 0 = no; ^*a*^Scale from 1 (totally disagree) to 7 (totally agree), ^*b*^Scale from 1 (not at all) to 5 (very much).

### Preliminary Analyses

To evaluate whether rumination about job insecurity could be distinguished from job insecurity, two models were compared: Model 1, a four-factor model in which job insecurity and rumination about job insecurity at T1 and T2 were estimated, and Model 2, a two-factor model, where job insecurity and rumination about job insecurity were combined and measured as one construct at T1 and at T2. Discriminant validity for job insecurity and rumination about job insecurity was demonstrated based on several findings. First, Model 1 fit the data significantly better than Model 2, Δχ^2^(3) = 44.16, *p* < 0.05. Second, in this model, a correlation of 0.61 (*p* < 0.05) was found between job insecurity and rumination about job insecurity at both time points, which shows that both constructs only have a shared variance of 37% (i.e., maximum shared variance [MSV] of 0.37). The correlation coefficient was well below the recommended cut-off of 0.85 (Weston and Gore, 2006; Kline, 2010), indicating good discriminant validity. In addition, we calculated the average variance extracted (AVE) for rumination about job insecurity: it had an AVE of 0.72 at T1 and 0.69 at T2. Also, according to the criterion of Fornell and Larcker (1981) (i.e., MSV < AVE), discriminant validity was thus established on the construct level. Finally, when testing the two-factor model (Model 2), the rumination about job insecurity items showed high factor loadings (>0.79) compared to the item originally measuring job insecurity (0.62); hence, the common factor explained more variance of the rumination about job insecurity items compared to the item from the job insecurity scale.

Based on Model 1, where job insecurity and rumination about job insecurity were modeled as two separate constructs at T1 and T2, we conducted further tests. To rule out that changes in the scale scores were the result of changes in the measurement model, we investigated whether the factor loadings of the rumination about job insecurity measurement model were invariant across time using longitudinal CFAs (see Table 2, upper part of the table). When the unconstrained model (Model 1a) was compared to the constrained model with factor loadings specified to be equal over time (Model 2a), the fit did not significantly decrease. Furthermore, fit did not decrease when item intercepts were set to be invariant (Model 3a). This indicates that the factor loadings and the item intercepts were equal over time and temporal effects in subsequent analyses represent true changes. To also ensure that the language difference (Dutch versus French) did not affect the results, three increasingly constrained models were tested in which certain measurement model parameters (i.e., factor loadings and item intercepts) were constrained to be equal across the two language groups (see Table 2, lower part of the table). All the necessary parameters (e.g., factor loadings and intercepts) in the measurement model were invariant between the Flemish- and the French-speaking group. More strict testing, such as the invariance of the residual variance, was not applied as it has previously been considered unreasonably strict and not relevant for the type of hypotheses investigated in this paper (Byrne and van de Vijver, 2010; Milfont and Fischer, 2010).

**TABLE 2:** Results of the invariance tests across T1 and T2 and across language (*N* = 699).

	*df*	χ*^2^*	CFI	SRMR	RMSEA		Model comparison		
							Δχ*^2^*	Δ*df*	Significant difference
**Invariance over time**									
(1a) Freely estimated model (configural invariance)	15	177.74*	0.95	0.05	0.125				
(2a) Factor loadings invariant (weak invariance)	17	182.84*	0.95	0.05	0.11	1a vs. 2a	5.10^a^	2	*ns*
(3a) Indicator intercept invariant (strong invariance)	21	191.09*	0.94	0.06	0.10	2a vs. 3a	8.24	4	*ns*
**Invariance across language**									
(1b) Freely estimated model (configural invariance)	30	210.91	0.94	0.06	0.12				
(2b) Factor loadings invariant (weak invariance)	34	215.32	0.94	0.06	0.12	1b vs. 2b	4.41	4	*ns*
(3b) Indicator intercept invariant (strong invariance)	38	220.64	0.94	0.06	0.11	2b vs. 3b	5.32	4	*ns*

*p < 0.05.

### Test of Hypotheses

To investigate Hypothesis 1, in which the lagged relationship between job insecurity and actual turnover was predicted, a cross-lagged model with this path was specified, χ^2^ (60) = 248.0, *p* < 0.001, CFI = 0.83, SRMR = 0.08, RMSEA = 0.06. In line with Hypothesis 1, a significant cross-lagged path could be detected (β = 0.27, *p* < 0.05).

**FIGURE 2:**
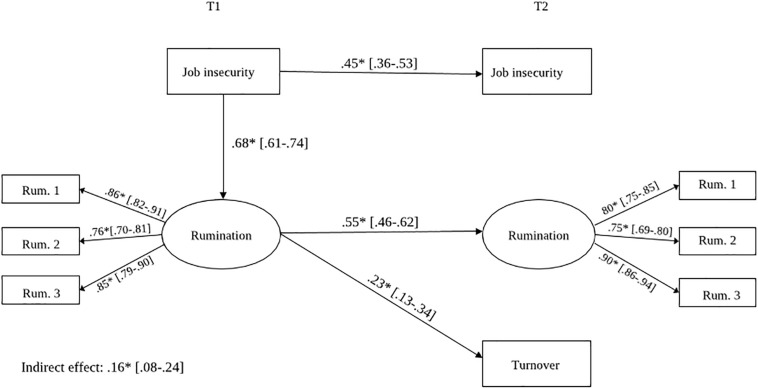
Sturctural model: standardized coefficients. Control variables, residuals, and relations between residuals are not depicted in this figure for readability reasons. ^∗^ < 0.0.5, Model fit: X^2^(60) = 247.83, *p* < 0.0001, CFI = 0.83, SRMR = 0.08, RMSEA = 0.06.

To investigate Hypothesis 2, a full mediation model with a path from job insecurity at T1 to rumination about job insecurity at T1, and from rumination about job insecurity at T1 to turnover at T2 was estimated, χ^2^ (60) = 247.83, *p* < 0.001, CFI = 0.83, SRMR = 0.08, RMSEA = 0.06 (see Figure 2). The path from job insecurity at T1 to rumination about job insecurity at T1 was significant (β = 0.68, *p* < 0.05), as well as the path from rumination about job insecurity at T1 to actual turnover at T2 (β = 0.23, *p* < 0.05). Adding a direct path from job insecurity at T1 to turnover at T2 to the full mediation model (i.e., partial mediation model) did not increase model fit, χ^2^(59) = 244.96, *p* < 0.001, CFI = 0.83, SRMR = 0.08, RMSEA = 0.06; Δχ^2^(1) = 44.16, *p* > 0.05. There was no direct relationship between job insecurity at T1 and turnover at T2 (β = 0.12, *ns*). Hence, we decided to select the more parsimonious full mediation model over the partial mediation model to formally test for mediation. In the full mediation model, the indirect effect of job insecurity at T1 on turnover at T2 via rumination about job insecurity at T1 was significant; the bias-corrected confidence interval for the indirect effect did not include zero (*z* = 0.16; *p* < 0.05; 95% CI [0.08,0.24]), which supported Hypothesis 2. Some of the model fit indicators were somewhat below the recommended cut-off point in the models tested; however, local fit indices were in line with the previous findings and theory in job insecurity research.

## Discussion

This study aimed to investigate the relationship between job insecurity, fear of losing the job, and actual turnover over a 1-year period. Even though turnover has frequently been studied indirectly using turnover intentions as a proxy (Berntson et al., 2010; Emberland and Rundmo, 2010; Staufenbiel and König, 2010), only a few studies have investigated the relationship between job insecurity and actual turnover, a detrimental outcome for organizations. According to our predictions, job insecurity was positively related to actual turnover 1 year later. So far, the majority of studies have relied on the assumption that employees’ intentions also translate into subsequent behaviors, which is dependent on a variety of different factors, weakening the strength of the predictive value of turnover intention (Cohen et al., 2015). Actual turnover is an important outcome that needs to be investigated because it implies that important organizational knowledge is lost. This particularly concerns knowledge and information gained through work experience at the particular organization that is often not documented. Turnover, in particular voluntary turnover, can also be a problem for organizations because employees who are more valuable and motivated may leave the organization first (Greenhalgh and Rosenblatt, 1984). As a result, the organization may end up with employees who either do not believe they can get another job or do not have a strong profile (Aronsson and Göransson, 1999). This may have severe consequences for the organizational performance, as the competitiveness of organizations today, with only small marginals between organizations, strongly depends on the creativity and knowledge of employees (Niesen et al., 2014).

In addition, this study made an important contribution to the job insecurity literature by investigating a new mechanism that explains the relationship between job insecurity and actual turnover. Previous studies on the explanatory mechanisms underlying the job insecurity–outcome relationship have either focused on sequences of outcomes or certain theoretical explanations. For instance, other outcomes of job insecurity have been presented as explanations of the relationship between job insecurity and turnover intentions, including occupational (Mauno et al., 2014) or general well-being (Stiglbauer et al., 2012), organizational support (Günalan and Ceylan, 2015), and work engagement (Metin Camgoz et al., 2016). Examples of theoretical explanations deriving from psychological theories that do not refer to other job insecurity outcomes are perceived control (Vander Elst et al., 2014c) and psychological contract breach (De Cuyper and De Witte, 2006). Rumination about job insecurity as an explanation of the aversive consequences of job insecurity has not been investigated previously. It is considered a strain-based mechanism indicating an intensification of the job insecurity experience. Specifically, we predicted rumination about job insecurity to mediate the lagged relationship between initial feelings of job insecurity and subsequent employee turnover. In line with this prediction, the results showed that, over time, job-insecure employees were more likely to change jobs because they started to ruminate about their potential job loss. Rumination about job insecurity may represent a strain-based mechanism: it may be conceived as the cognitive activity focusing on repetitive thoughts about a potential job loss as a response to the initial fear of job insecurity. In line with COR, job-insecure employees experience a stress reaction and may attempt to find a solution to the eventual job loss, resulting in rumination about job insecurity. Rumination about job insecurity depletes resources further, and employees might leave the organization in order to preserve their resources or get dismissed because they can no longer perform to the standards expected of them. So far, mediation by rumination about job insecurity in the job insecurity-outcome relationship has not been studied. However, our results demonstrate that it is an important factor that can help in understanding how job insecurity develops and results in, for example, turnover. Our finding corresponds to the results of studies in which other strain-related mechanisms, such as occupational well-being, were investigated. Mauno et al. (2014), for example, demonstrated that reduced employee well-being (i.e., an indication of strain) mediated the relationship between job insecurity and turnover intentions. Furthermore, concentration was found to be a mediator for the relationship between job insecurity and martial functioning (Barling and Macewen, 1992). Compared to the existing studies on strain-based mechanisms (Barling and Macewen, 1992; Mauno et al., 2014), our study addresses a more job insecurity-specific mechanism, and by being so specific, it might also be better suited to understand the job insecurity–consequences relationship.

Even though rumination was found to be one important mediator in the relationship between job insecurity and turnover, other mediators should be tested. Indeed, although we did not find evidence for a direct relationship between job insecurity at T1 and turnover at T2 after controlling for rumination about job insecurity at T2, the indirect effect via rumination about job insecurity accounted for only 59%. The effects of job insecurity may thus be explained by other mechanisms as well, such as psychological contract breach, lack of perceived control, and threat to the benefits of work (Selenko and Batinic, 2013; Vander Elst et al., 2014a). Therefore, future studies should consider investigating multiple mediators in addition to rumination about job insecurity. It is important to investigate which mechanisms are most important so that organizations can focus on these mechanisms to prevent job insecurity from resulting in negative outcomes.

### Limitations and Future Studies

One limitation of this study is the operationalization of employee turnover, in which no distinction was made between voluntary and involuntary turnover. Different processes might, however, account for the relationships between job insecurity and either voluntary or involuntary turnover (e.g., withdrawal versus bad performance as a result of strain). In addition, the question of whether job insecurity is related to voluntary and/or involuntary turnover may have important implications for organizations. For organizations, it might be particularly detrimental if highly motivated employees with a key position leave voluntarily, whereas the involuntary turnover of less motivated workers might have the opposite effect (cf. Greenhalgh and Rosenblatt, 1984). Even though we introduced rumination about job insecurity as a process that accounts for the relationship between job insecurity and both voluntary and involuntary turnover, future studies should further investigate the relationships of job insecurity with both types of turnover, as well as the mechanisms underlying these relationships. Additionally, in this study, only external turnover was studied. Future studies should also focus on internal turnover, where employees change jobs within the organization.

In addition, we used a one-item measure of job insecurity due to practical considerations and time constraints. Compared to job insecurity scales, the psychometric properties of single items cannot be determined, and it is harder to capture multi-faceted constructs (Fisher and To, 2012). However, using single items to investigate job insecurity is not uncommon and has led to fruitful research (e.g., Anderson and Pontusson, 2007; Burchell, 2009; Erlinghagen, 2009). Moreover, the item used in this study has been included in many job insecurity scales that are widely used today (e.g., Vander Elst et al., 2014b) and may therefore represent the core of the concept of affective job insecurity. The meta-analysis by Sverke et al. (2002) on the effects of one versus multi-item measurements of job insecurity indicated that one-item measurements tend to underestimate the relationship between job insecurity and potential outcomes. Single job insecurity items seem to grasp a smaller portion of the variance of the conceptual domain of job insecurity compared to job insecurity scales. As we found statistically significant relationships between the variables under investigation, we can speculate that these relationships might be even stronger when a multiple-item measurement of job insecurity is used. Future researchers might therefore benefit from trying to replicate our results using a validated job insecurity scale (e.g., Hellgren et al., 1999; Vander Elst et al., 2014b). They should also include the cognitive component of job insecurity—that is, the likelihood of job loss—because in this study, job insecurity only concerned the worry about potential job loss (affective job insecurity; Hartley et al., 1991).

The data included two time waves with a 1-year time lag. This made the investigation of mediation difficult. We used a time lag of 1 year to adjust for seasonal fluctuations, as well as to consider the cycle of work-intensive periods of organizations. One year might, however, not be the optimal time lag to study the development of job insecurity and its relationship with rumination and turnover, and this may have resulted in an underestimation of the relationships (Ford et al., 2014). A shorter time period might have been more appropriate, particularly to capture the full strength of the relationship between job insecurity and rumination (cf. Ford et al., 2014). Therefore, we decided to model the mediation and in particular the relationship between job insecurity and rumination about job insecurity cross-sectionally. Even though more and more studies on job insecurity are using a longitudinal design, still relatively little research has been conducted on how job insecurity develops over time into the negative consequences of which we are well aware (Sverke et al., 2002). Studies using different time lags may clarify the relationships of job insecurity with rumination about job insecurity and turnover further.

Moreover, future studies investigating the development of the negative consequences of job insecurity should investigate other causal relationships from rumination about job insecurity to job insecurity, such as a reciprocal relationships. When employees gain new insights, which may indicate that their job might not be in danger, for instance through information from the organization, they may shift (back) from rumination about job insecurity to initial fear of job loss. Job insecurity experiences are subjective appraisals of the working context, which is, among other things, influenced by the feelings and perceptions of the individual (Sverke et al., 2002; De Witte and Näswall, 2003). Moreover, rumination about job insecurity might affect actual performance. Reduced performance might increase the risk of involuntary turnover, as employees may not live up to the standards of the organization, which in turn might affect job insecurity (cf. Niesen et al., 2014). Investigating this dynamic process of job insecurity and rumination about job insecurity is in line with the spiral loss hypothesis proposed by COR. This hypothesis suggests that individuals drained of resources are more vulnerable to further resource loss due to the close connection between resources, as well as to the fact that individuals do not have enough resources to prevent future resource loss (Hobfoll, 1989, 2001). To investigate a loss spiral between job insecurity and rumination about job insecurity further, future studies should look more closely into the absolute values of job insecurity and rumination about job insecurity and how they develop over time and in relation to each other.

The retention rate in this study was rather low. One reason for this might be that employees who participated at T1 were asked if they would like to receive results from the data collection and consented by this to receive information about and invitations to new data collections. However, they might not have been motivated to participate in a follow-up study 1 year later. In addition, the e-mail addresses that respondents provided to get the information about the study results might no longer have been correct or in use. Individuals might have changed jobs within the organization or been employed by another organization, resulting in another e-mail address, or there might have been spelling mistakes in the e-mail address.

Next, future studies might also benefit from investigating the function of rumination. In general, rumination has been studied as a destructive process that depletes the individual (cf. Lyubomirsky et al., 1998). This conceptualization corresponds to our reasoning regarding involuntary turnover: individuals who ruminate and, through their rumination about a potential job loss, are drained might not be able to fulfill the expectations at work and thus get fired. However, some scholars have also acknowledged that rumination can result in problem-focused coping reactions, by which individuals try to find a solution to the problematic situation that caused the rumination process (Querstret and Cropley, 2012). Job-insecure individuals may, for instance, use their rumination process in a constructive way, in which they decide to look for another, more secure position and leave the organization. In addition, the mechanisms behind rumination might be important to investigate: stress complaints, for instance, could be an explanatory factor through which rumination is associated with, for instance, performance.

### Theoretical and Practical Implications

This study has important practical implications for organizations. Because job insecurity is related to employee turnover over time, organizations should work more actively to prevent the initial fear of losing the job (De Witte et al., 2015). Clear and transparent communication about organizational changes is one way to prevent experiences of job insecurity (Jiang and Probst, 2013). Additionally, employee participation in decision-making can be implemented during restructurings to increase employees’ feelings of control, as well as to keep them informed on what is going on.

Another important step is to prevent employees from ruminating about job insecurity. Effective strategies for reducing rumination in general may concern placing priority on leisure time and acknowledging leisure time as an important time to recover. Individuals who ruminate a lot have been found to plan their leisure time differently: they, for instance, use their leisure time more passively, which is not as effective for recovery (Cropley and Millward, 2009). Organizations could provide employees with the opportunity to participate in workshops and inform them about the importance of leisure time and of keeping a good balance between work and non-work life. Acceptance and commitment therapy, where mindfulness is one key factor, have lately been introduced into workplaces (Seear and Vella-Brodrick, 2012). In such therapy, employees learn how to live in the moment instead of stressing about past or potential future events. This may prevent them from ruminating about their job in the future.

## Conclusion

To conclude, this study investigated the prospective relationship between job insecurity and actual turnover, an outcome that has been rarely studied even though it is an essential outcome that affects organizational functioning and efficiency. So farm turnover intention has been primarily studied as a proxy for actual turnover, assuming that employees’ intentions also translate into subsequent behaviors. In line with our predictions, a positive prospective relationship between job insecurity and actual turnover was found. In addition, rumination about job insecurity was investigated as a new strain-based mechanism that explains the relationship between job insecurity and actual turnover. In line with our prediction, job-insecure employees were more likely to change jobs because they started to ruminate about their potential job loss.

## Data Availability Statement

The datasets generated for this study are available on request to the corresponding author.

## Ethics Statement

Ethical review and approval was not required for the study on human participants in accordance with the local legislation and institutional requirements. The patients/participants provided their written informed consent to participate in this study.

## Author Contributions

AR, TV, and HD: conceptualization, methodology, formal analysis, writing – original draft preparation, and contributed significantly to the manuscript. AR: lead role. TV: writing – review and editing. HD: supervision, project administration, and funding acquisition.

## Conflict of Interest

The authors declare that the research was conducted in the absence of any commercial or financial relationships that could be construed as a potential conflict of interest.

## Footnotes

1 Vacature is a weekly published HR magazine offering career information as well as vacancies to readers and Internet users. It mainly addresses highly educated employees.
